# Single-cell assembled genomes predict enhanced bacterial metabolic cross-feeding potential in carbon-enriched soils

**DOI:** 10.1093/ismejo/wrag071

**Published:** 2026-03-27

**Authors:** Hanyue Guo, Qicheng Xu, He Zhang, Yizhu Qiao, Yang Song, Yinghua Duan, Ning Ling, Qirong Shen

**Affiliations:** Jiangsu Provincial Key Lab for Organic Solid Waste Utilization, National Engineering Research Center for Organic-based Fertilizers, Jiangsu Collaborative Innovation Center for Solid Organic Waste Resource Utilization, Nanjing Agricultural University, Nanjing, Jiangsu 211800, China; Jiangsu Provincial Key Lab for Organic Solid Waste Utilization, National Engineering Research Center for Organic-based Fertilizers, Jiangsu Collaborative Innovation Center for Solid Organic Waste Resource Utilization, Nanjing Agricultural University, Nanjing, Jiangsu 211800, China; Jiangsu Provincial Key Lab for Organic Solid Waste Utilization, National Engineering Research Center for Organic-based Fertilizers, Jiangsu Collaborative Innovation Center for Solid Organic Waste Resource Utilization, Nanjing Agricultural University, Nanjing, Jiangsu 211800, China; Jiangsu Provincial Key Lab for Organic Solid Waste Utilization, National Engineering Research Center for Organic-based Fertilizers, Jiangsu Collaborative Innovation Center for Solid Organic Waste Resource Utilization, Nanjing Agricultural University, Nanjing, Jiangsu 211800, China; Plant–Microbe Interactions, Institute of Environmental Biology, Department of Biology, Science4Life, Utrecht University, Padualaan 8, Utrecht, 3584 CH, The Netherlands; Plant–Microbe Interactions, Institute of Environmental Biology, Department of Biology, Science4Life, Utrecht University, Padualaan 8, Utrecht, 3584 CH, The Netherlands; State Key Laboratory of Efficient Utilization of Arid and Semi-arid Arable Land in Northern China / Institute of Agricultural Resources and Regional Planning, Chinese Academy of Agricultural Sciences, Beijing 100081, China; Jiangsu Provincial Key Lab for Organic Solid Waste Utilization, National Engineering Research Center for Organic-based Fertilizers, Jiangsu Collaborative Innovation Center for Solid Organic Waste Resource Utilization, Nanjing Agricultural University, Nanjing, Jiangsu 211800, China; Centre for Grassland Microbiome, State Key Laboratory of Grassland Agro-ecosystems, College of Pastoral Agricultural Science and Technology, Lanzhou University, Lanzhou, Gansu 730020, China; Jiangsu Provincial Key Lab for Organic Solid Waste Utilization, National Engineering Research Center for Organic-based Fertilizers, Jiangsu Collaborative Innovation Center for Solid Organic Waste Resource Utilization, Nanjing Agricultural University, Nanjing, Jiangsu 211800, China

**Keywords:** single-cell sequencing, carbon availability, fertilization, metabolic interactions

## Abstract

Carbon availability is a key determinant of soil microbial community structure and function, shaping their metabolic activities and interactions. However, the mechanisms driving these interactions and their ecological and evolutionary implications remain poorly understood. Here, we integrated single-cell Cell Sorting and Sequencing (scCS-seq) technologies with community metabolic modeling to investigate the genomic traits and metabolic interactions of microorganisms in soils with different carbon availability. We find that microorganisms in carbon-enriched soils exhibit larger genomes with more coding sequences and enrichment of biosynthesis-related CAZyme families (e.g. GT83, GT44), whereas those in carbon-depleted soils adapt to resource scarcity with streamlined genomes and higher GC content. Our metabolic modeling predicts a stronger potential for cross-feeding in carbon-enriched soils, with amino acids and aromatic compounds identified as preferentially exchanged metabolites. This enhanced cross-feeding potential may promote resource sharing and functional complementarity among soil microorganisms. These findings highlight the role of microbial metabolic interactions as fundamental drivers of community assembly and ecosystem functioning, providing new insights into the ecological and evolutionary principles that structure soil microbiomes.

## Introduction

Soil is one of the most complex ecosystems on Earth, harboring an immense diversity of microorganisms [1]. These soil microbes play a critical role in the global carbon cycle by decomposing organic matter, transforming nutrients, and regulating energy flow, thereby maintaining the stability and productivity of ecosystems [2, 3]. Understanding the genetic diversity of soil microbes and their responses to environmental changes is essential for improving ecosystem functionality and resilience [4, 5].

Although cultivation-based approaches and environmental DNA sequencing have advanced our understanding of soil microbial diversity, they remain insufficient for resolving strain-level heterogeneity and metabolic interactions [6, 7]. Metagenomics enables reconstruction of community metabolic potential but typically produces metagenome-assembled genomes (MAGs) that represent composite or averaged populations, often obscuring rare taxa and fine-scale functional variation. In contrast, single-cell genome sequencing provides direct access to genomes from individual microbial cells, preserving strain-level diversity and enabling more accurate linkage between phylogeny and metabolic potential. Here, we envision a “single-cell Cell Sorting and Sequencing” (scCS-seq) strategy, an innovative approach that shifts the focus from community-level assemblies to individual microbes [8, 9]. The scCS-seq technique allows for comprehensive analysis of microbial genomes, enabling the acquisition of genomic information from individual cells that closely reflect the characteristics of the original organisms [10, 11]. By integrating single-cell genomics with metagenomic data, it is possible to quantify the abundance of individual lineages and infer microbial co-occurrence and metabolic interaction networks at unprecedented resolution. This strategy thus holds immense potential for advancing our understanding of microbial diversity, population genetics, and evolutionary dynamics, as well as for illuminating microbial interactions at the metabolic level [4, 12].

Microbial co-occurrence networks have been widely used to infer community structure and ecological relationships [13–15]. However, these methods have limitations in fully capturing the complexity of microbial ecosystems. By integrating metagenomic data with single-cell genomics, we can quantify the abundance of individual microbes within a community and enhance our understanding of co-occurrence patterns [11, 16]. This integrated approach offers a powerful framework for identifying potential metabolic cross-feeding interactions and understanding how microbial communities organize and stabilize through metabolic interdependencies [17]. Although recent studies have employed this combined strategy to investigate microbial ecosystems in marine [11], wastewater [18], and human gut [19], there is limited research on complex environmental ecosystems such as soil.

Long-term fertilization is a major driver of soil carbon availability and microbial community structure [2, 3]. Long-term chemical-only fertilization, which are commonly used in conventional agriculture, can create “carbon-depleted” environments (C-depleted), limiting microbial growth and activity [20, 21]. In contrast, long-term organic fertilization can increase soil organic matter, providing microbes with diverse carbon sources and promoting a more “carbon-enriched” environment (C-enriched) [2]. Although extensive research has been conducted on microbial community characteristics in soils with different carbon availability (e.g. C-depleted and C-enriched) [22, 23], there is still limited understanding of the metabolic interaction mechanisms at the single-cell scale and their associated ecological and evolutionary impacts. Previous research has shown that C-enriched soils provide readily available carbon substrates, thereby stimulating microbial growth and increasing community biomass [24, 25]. Nutrient-rich conditions can promote metabolite overflow and niche complementarity, ecological mechanisms that are known to favor the evolution of cross-feeding and syntrophic interactions among microorganisms [26]. Therefore, we hypothesize that in C-enriched soils, microorganisms harbor more biosynthesis-related genes and exhibit more complex metabolic networks with stronger metabolic cross-feeding dependencies.

Here, we collected soil samples from four long-term field experiments across different regions of China, where soils had been treated with either chemical-only fertilizers or organic-only fertilizers continuously for ~40 years, resulting in C-depleted and C-enriched soils, respectively. Using scCS-seq technology, we characterized the genomic and functional features of individual microbes within soils with contrasting carbon content. By integrating metagenomic data with single-cell genomic analysis, we aimed to uncover the biological interactions (co-occurrence networks) among soil bacteria. We further reconstructed genome-scale metabolic models to predict cross-feeding interactions within co-occurring microbial consortia. We further validated key model-predicted metabolic interactions using co-cultivation experiments coupled with LC–MS metabolomics, providing experimental support for the inferred metabolic exchange patterns. This integrative framework combining ecosystem-scale meta-omics data with scCS-seq and metabolic modeling offers a more comprehensive understanding of microbial interactions that support crop yield improvement and agricultural sustainability.

## Materials and methods

### Field sites and soil sample collection

We selected four long-term fertilization experiment sites located in major grain-producing regions across China (Table S1). Each of these field experiments has been running for ~40 years. The soils involved in this study subjected chemical and organic fertilization, respectively, resulting in different carbon availability (Table S1): carbon-depleted (C-depleted) and carbon-enriched soils (C-enriched). For each treatment, three separate plots were established at each experimental site, with all plots arranged in a randomized block design. In each plot, 10 soil cores, each with a diameter of 5 cm, were collected from a depth of 0 to 20 cm and combined to create a composite sample representative of the plot. All soil samples DNA was extracted using the FastDNA SPIN Kit for Soil (MP Biomedicals) and metagenomic sequencing was performed on a NovaSeq 6000 System (Illumina).

### Acquisition of single-cell of soil microbiome in single-cell cell sorting and sequencing

#### Sample preparation for profiling soil

Approximately 5 g of soil was suspended in 45 ml of Tween 20 (T20, 0.5% in PBS buffer). All buffers were sterilized by autoclaving prior to use. The suspension was homogenized with a blender for 3 min. After dispersion, 20 ml of the soil slurry was carefully layered onto 18 ml of 80% Nycodenz in a sterile, enzyme-free 50 ml centrifuge tube. The mixture was centrifuged at 15 000 × g for 40 min at 4°C with slow acceleration and deceleration [27]. Following centrifugation, three distinct layers were observed: a top layer containing T20, a middle layer enriched in microbial cells, and a bottom layer containing Nycodenz and soil particles. The cell layer above the Nycodenz was carefully collected with a pipette and resuspended in 5 ml of PBS buffer to obtain the soil microbial cell suspension for subsequent experiments [27].

#### Isolation of individual microbe from the soil sample

The single-cell sorting process was conducted using the CellenONE system (Cellnion, France), and all single-microbe were obtained through this platform. For scCS-seq, the extracted soil microbial cell solution was diluted with sterile deionized water for performing the subsequent single-cell sorting. The diluted cell solution was then sorted using the CellenONE system. Specifically, the sorting needle of CellenONE takes a diluted sample of cells and stored it in the sorting needle. Then the sorting needle ejected the cells in the “safe zone” (the range set determined by a high-resolution microscope ensuring that there is only one cell in the ejected droplet) at picoliters. The mechanical moment arm sorted the droplets containing individual cells into the bearing plate with lysis buffer.

#### Multiple displacement amplification for single-cell genome sequencing

Each single cell was lysed in 3 μl of lysis buffer for 10 min at 65°C, followed by the addition of 3 μl of termination buffer to neutralize the reaction (CW2843, CWBIO, China). Reaction buffer and DNA polymerase were then added according to the protocol (CW2843, CWBIO, China), and the mixture was incubated at 30°C for 4 h with 30°C hot-lid temperature for multiple displacement amplification (MDA) reactions. A blank control (without cells) was included to detect and quantify potential contamination. Finally, the MDA products were purified by Agencourt AMPure XP Beads (CW2508, CWBIO, China).

#### Library construction and sequencing of the single cells

For each single cell, 0.2 μg of DNA was used as input for the DNA library preparation. Sequencing libraries were prepared using the CWseq Universal DirectFast DNA Library Prep Kit (Illumina & MGI) DNA Library Prep Kit for Illumina (CW3048, CWBIO, China), with index codes assigned to each sample. Briefly, genomic DNA was fragmented into ~300 bp pieces by enzymatic digestio, followed by end polishing, A-tailing, and ligation with full-length adapters for Illumina sequencing. The fragments then underwent PCR amplification. The PCR products were purified using AMPure XP (CW2508, CWBIO, China), and the DNA concentration was measured with a Qubit 3.0 Fluorometer (Invitrogen, USA). The DNA libraries were sequenced using 150 bp paired-end reads on a NovaSeq 6000 System (Illumina).

### Sequencing data analysis

#### Analysis of metagenomic data

Data quality was assessed using FastQC v0.11.9 [28], and low-quality sequences and adaptors were removed with Trimmomatic v0.38 (average quality cutoff = 20) [29]. Assembly of clean reads was performed using Megahit v1.2.9 with k-mer sizes ranging from 21 to 141. Open reading frames were predicted with Prodigal v2.6.1. SAGs (single amplified genome) were then quantified in these environmental samples by mapping the cleaned metagenomic reads to assembled genomes using CoverM v0.7.0.

#### Analysis of bacterial single-cell genome sequencing data

To ensure the accuracy and reliability of the SAG, we performed extensive quality control checks at various stages of the analysis. Reads that passed illumina’s chastity filter were first quality checked using FastQC [28], and then quality trimmed using Trimmomatic v0.38 for each sample [29]. To identify and remove potential contaminant DNA fragments introduced during MDA or library preparation, all quality-filtered reads were taxonomically classified using Kraken2 [30], enabling the detection and exclusion of misassigned or non-target reads prior to assembly. Clean reads were assembled using SPAdes v3.15.0 in single-cell mode with multiple k-mer sizes (21–121) to mitigate MDA-induced coverage bias and chimeric artifacts [31]. We assessed the quality of each SAG using CheckM [32]. After filtering out low-quality genome (completeness <50% and contamination >10%), medium-high quality genomes were retained. We recalculated the estimated genome size for each SAG using the following formula: Genome size = assembly length / CheckM-estimated completeness. We took a conservative approach by removing any unusually large genomes from the dataset during the subsequent analysis steps. The taxonomic annotation and phylogenetic tree were constructed based on the Genome Taxonomy database Release 207 (GTDB) using GTDB-Tk v1.5.0 with “de_novo_wf” module [33]. We used Prokka to predict ORFs in SAGs [34]. To estimate the functions related to organic C metabolisms, the ORFs were annotated against the dbCAN2 database using hmmscan-parser.sh (https://bcb.unl.edu/dbCAN2/download/Databases/dbCAN-old@UGA/) with default parameters. Functional distance is defined as the Jaccard distance calculated from a presence/absence matrix of CAZy annotations.

#### Joint analysis of single-cell and metagenomic data

To investigate how SAG-communities establish complex structures and functions through ecological interactions, we employed an integrative strategy that combined SAG information with metagenomic sequencing data from environmental samples. This approach not only enables the identification and characterization of functional potential at the community level, but also allows quantification of individual SAG at strain-level resolution, thereby facilitating comparisons of abundance dynamics across treatments and the inference of potential microbial association networks. The abundance of each SAG was calculated using a mapping-based strategy. Specifically, the abundance of each SAG was determined by mapping environmental metagenomic clean reads to SAG using CoverM v0.7.0 with a 100% identity threshold and default multi-mapping handling [35].

#### Genome metabolic modelling and cross-feeding interaction predictions

We integrated genomic information (SAG abundances) to infer soil microbial association networks, aiming to explore how microbial communities form complex structures and functions through interactions. To account for compositional effects in microbial abundance data, we analyzed the SAG-based abundance table across samples using SparCC, retaining only correlations with *P* < .05 and |r| > 0.6 for network construction. Different modules in the network are considered as a sub-SAG community [36]. The number of nodes in a sub-community is the size of the sub-SAG community.

The genomes were processed using CarveMe v1.2.2 to reconstruct individual metabolic models [37]. CarveMe was run without gap-filling (--nogapfill parameter) using the IBM CPLEX solver. The primary reason for this choice was to avoid predicting potential false-positive cross-feeding interactions that could arise from artificially adding reactions to incomplete genomes. Models were automatically pruned by CarveMe to remove dead-end reactions and ensure metabolic functionality. Reconstructed metabolic models of these genomes were then analyzed using SMETANA v1.2.0 to predict putative metabolic cross-feeding interactions [38]. SMETANA was run in detailed modes with the solver IBM CPLEX v12.10, using the default media provided by the software package (minimal media). The SMETANA sum score (sum of the SMETANA scores in a given sub-community) is employed as a community-wide version of the pairwise SMETANA score. The SMETANA score, integrating several metrics to estimate the metabolic dependencies within a given community, was used to evaluate the probability of each potential cross-feeding interaction among genome communities [5, 38]. To compare communities of different sizes, the SMETANA sum score (calculated by summing all predicted SMETANA scores for a given sub-community) was normalized by dividing it by the total number of SAGs in a given sub-community. We referred to this new score as normalized smetana score [5]. To categorize the different metabolites in the SMETANA database (e.g. amino acids), we mapped the metabolite identifiers to the BIGG database (http://bigg.ucsd.edu/).

#### Co-culture setup and LC–MS metabolomic analysis

To validate the exchange metabolites and cross-feeding interactions predicted by the SMETANA model, we designed a co-cultivation experiment followed by metabolomic analysis. Specifically, we selected two microbial communities from the co-occurring networks: the C-enriched group and the C-depleted group. All bacterial strains used in this study were co-occurring SAG representatives. Detailed information about the strains and their isolation methods can be found in the supplementary materials. In the co-cultivation experiment, all individual strains from the C-enriched group were mixed together with equal OD_600_ and volume and then cultured in the M9 minimal medium supplemented with glucose as the sole carbon source. Similarly, all individual strains from the C-depleted group were co-cultured in the M9 medium. Both co-cultures were maintained under sterile conditions and incubated at 37°C for 24 h. Each co-cultivation experiment was performed in triplicate to ensure reproducibility.

After the co-cultivation period, the supernatant from each co-culture was carefully collected by centrifugation (10 000 × g, 10 min) and filtered through a 0.22 μm membrane to remove any microbial biomass. The filtered supernatant was then used for metabolite extraction. Non-targeted metabolomics was performed using LC–MS. Detailed methods for sample preparation and LC–MS analysis are provided in the supplementary materials. The LC–MS data were used to identify and quantify the metabolites in the supernatants of the C-enriched group and the C-depleted group co-cultures. The data were processed using MetaboAnalyst software for peak detection, alignment, and normalization.

To assess metabolite differences between the C-enriched and C-depleted groups, we first calculated the SMETANA values for each metabolite. We then performed a Kruskal-Wallis test to compare the SMETANA values between the groups. For metabolites whose SMETANA values were significantly higher in one treatment group compared to the other, we interpret this as an indication of greater predicted cross-feeding potential under the corresponding soil environmental conditions. To validate these predictions, we used LC–MS to determine the abundance of metabolites in the two groups of co-cultures. The abundance of significantly predicted metabolites was compared between the two groups using the Kruskal-Wallis test to verify consistency with the SMETANA predictions.

#### Statistical analyses

To minimize the potential confounding effects arising from between-site heterogeneity, geographic variation was explicitly accounted for in our statistical analyses. Specifically, given the broad spatial distribution of the sampling locations, we applied a linear mixed-effects model in which carbon status was treated as a fixed effect, and sampling site (four locations) was included as a random effect. To account for potential confounding effects of soil properties, we performed phylogeny-informed PGLS models and variation partitioning analysis, with soil pH and available phosphorus (AP) included as covariates. For comparing differences in genome size, CDS counts, GC content, and size-normalized SMETANA scores, we used the Kruskal-Wallis test. We applied the Benjamini-Hochberg (BH) method to control the false discovery rate (FDR) for multiple comparisons. For CAZyme enrichment analysis, CAZyme annotations obtained from SAGs were converted into a presence-absence matrix for each CAZyme family across genomes, and differential analysis between C-enriched and C-depleted treatments was performed using DESeq2. To validate the topological significance of our networks, we implemented a null model analysis. We generated 1000 node-count-matched random networks for each treatment and compared the modularity of our empirical networks against these null distributions. To assess whether differences in network properties were influenced by unequal sample sizes, we performed a rarefaction-based resampling analysis. Specifically, 1000 rarefaction iterations were conducted, in which 18 single-cell genomes were randomly selected from each of the C-enriched and C-depleted groups, and co-occurrence networks were reconstructed using identical parameters.

## Results

### Features of soil bacterial single amplified genomes

**Figure 1:**
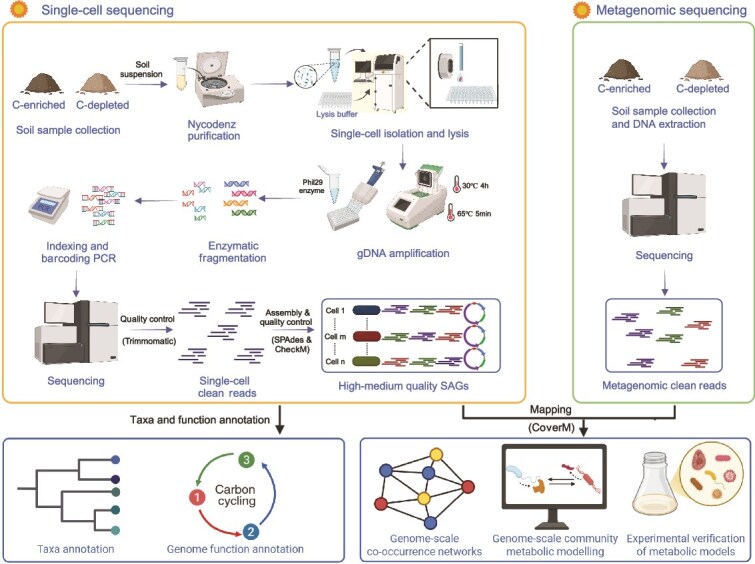
Workflow of single-cell sorting and sequencing (scCS-seq). The workflow illustrates a research framework combining single-cell sequencing with metagenomic sequencing to analyze the genomic characteristics and metabolic interactions of soil microorganisms. The section on the left details the steps of single-cell sequencing: soil suspension is extracted from soil samples and purified using Nycodenz. Single cells are then isolated and lysed, followed by amplification of their genomic DNA (gDNA) using Phi29 polymerase. The amplified DNA undergoes enzyme fragmentation and PCR barcoding for high-throughput sequencing. After quality control, medium-high quality single-cell amplified genomes (SAGs) are generated. These data are then used for species classification annotation and functional genome annotation. The section on the right shows the metagenomic sequencing process, where soil samples are subjected to metagenomic sequencing to obtain clean reads. These clean reads are used for quantifying SAG abundances. At the bottom, the section demonstrates the integrated analysis method that combines single-cell sequencing and metagenomic sequencing data. By analyzing genome-scale community composition, co-occurrence networks, and metabolic models, and experimentally validating these models, the framework serves to elucidate the driving factor and metabolic interactions within microbial communities.

**Figure 2:**
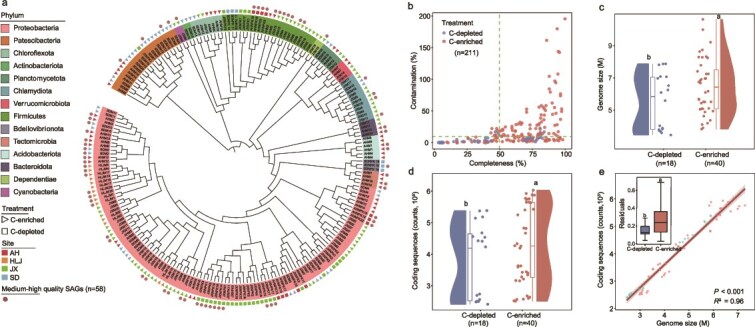
Features of soil bacterial single amplified genomes (SAGs). (a) The phylogenetic tree illustrates the taxonomic distribution of SAGs (n = 211) across different treatments. The color-coded outer ring represents different phyla. And the associated symbols indicate the carbon availability (C-depleted or C-enriched) soils and the sampling sites (AH, HLJ, JX, SD). (b) Genome completeness and contamination. Each point represents a SAG colored by the treatment. Medium-high quality SAGs (n = 58) typically show high completeness (>50%) and low contamination (<10%). (c) The comparison of genome sizes between SAGs from the C-depleted and C-enriched soils. Genome size = assembly length / CheckM-estimated completeness. (d) The distribution of coding sequences (CDS) among SAGs in the C-depleted and C-enriched soils. (e) A linear regression of genome size against the number of coding sequences in the two carbon availability soils. The boxplot compares the residuals of coding sequences between C-depleted and C-enriched soils.

To develop a comprehensive catalogue of soil bacterial single amplified genomes (SAGS), we assembled SAGs from bacterial single-cell isolates sourced from C-depleted and C-enriched soils (Fig. 1). A total of 211 species-level representative genomes were catalogued in our integrated database of soil bacterial genomes (Fig. 2a). The completeness and contamination for each SAG are shown in Fig. S1 and Table S2. And then we constructed a phylogeny that identified 14 distinct phyla, including well-represented groups in soil environments such as *Proteobacteria, Firmicutes*, and *Actinobacteriota* (Fig. 2a). We focused exclusively on medium-to-high quality SAGs with a completeness of ≥50% and contamination levels ≤10% (Fig. 2b). Among these SAGs, 40 medium-to-high SAGs were derived from the C-enriched soils and 18 SAGs from the C-depleted soils. Here, both the genome size and the coding sequences in SAGs from C-enriched soils were significantly greater than those from C-depleted soils, by 18% and 13%, respectively (Fig. 2c and d). To exclude phylum-level compositional effects, we conducted within-phylum comparisons for *Proteobacteria* and *Firmicutes*. Larger genome sizes under C-enriched conditions were consistently observed within both phyla (Fig. S2). At the genomic level, the GC content of SAGs in C-depleted soils were significantly higher than that of SAGs in C-enriched soils by 9% (Fig. S3). These results collectively suggest that carbon availability exert significant effects on the microbial community composition, genome characteristics, and functional potential. Larger genomes were found to contain more coding sequences (Fig. 2e). To further investigate the relationship between genome size and coding capacity, we performed a linear least-squares regression on a log–log scale and compared the distribution of residuals, which represent the difference between the actual and expected y-axis values (coding sequences). The residuals in the C-depleted soils were smaller compared to those in the C-enriched soils (Fig. 2e). This suggests that microbial genomes in C-depleted soils conform more closely to the expected genome size-to-coding sequence ratio, reflecting a more streamlined and efficient genomic organization.

### Functional traits of SAGs in different fertilized soils

**Figure 3:**
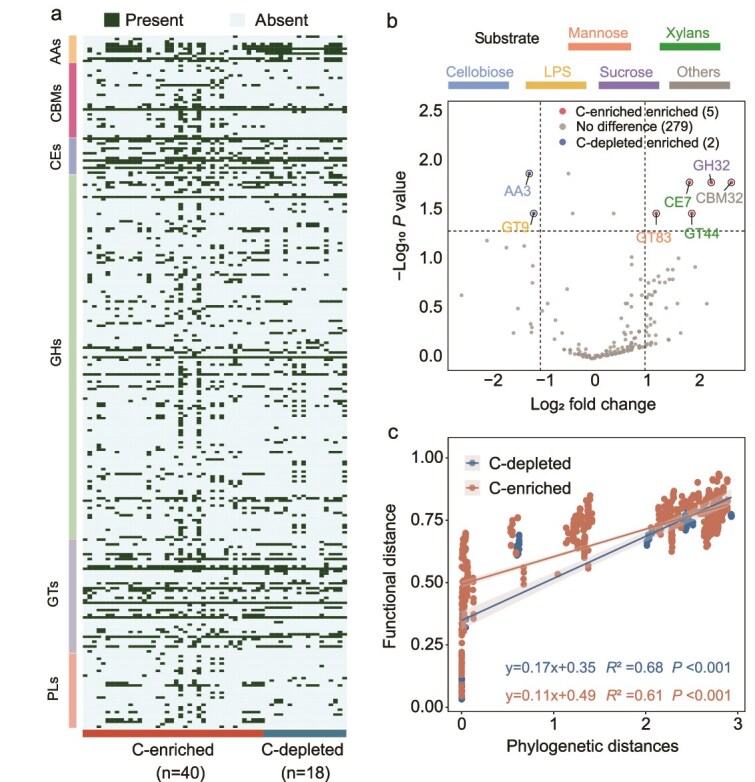
The genomic functional potential of SAGs. (a) The heatmap showcases the presence and absence of various carbohydrate-active enzymes (CAZymes) in different SAGs (n = 58), categorized into auxiliary activities (AAs), carbohydrate-binding modules (CBMs), carbohydrate esterases (CEs), glycoside hydrolases (GHs), glycosyl transferases (GTs), and polysaccharide lyases (PLs). (b) The volcano plot shows the CAZymes genes with significant differences in SAGs between the two carbon availability soils. The x-axis represents the log_2_ fold change, and the y-axis shows the negative logarithm of the p-value. The significance thresholds are shown as horizontal lines (false discovery rate (FDR) ≤0.05) and vertical lines (|log_2_ fold change| ≥1). Points on the left side of the plot (in blue) represent genes that are enriched in SAGs from C-depleted, whereas those on the right (in red) are enriched in SAGs from C-enriched. The color of the gene font represents the substrate type. (c) A correlation of phylogenetic distances and functional distance. Different colored lines represent different carbon availability soils.

To investigate the functional properties of the SAGs, we annotated their genes using the CAZymes (Carbohydrate-Active enZymes) database. From the genes annotation results, we derived the presence and absence of carbohydrate-active enzymes for each SAG (Fig. 3a). Heatmaps showing detailed CAZyme features of all SAGs are presented in Fig. S4. We further identified genes that were enriched in both kinds of soils (Fig. 3b). In C-depleted soils, AA3 (auxiliary activity) and GT9 (glycosyl transferases) genes were enriched, primarily utilizing cellobiose and lipopolysaccharide (LPS) as substrates/acceptor molecule, respectively. Conversely, enzyme families enriched in C-enriched soils included GT83, GT44, CE7, CBM32 and GH32 (glycoside hydrolase), which are commonly associated with mannose, xylan and sucrose metabolism.

We observed a positive correlation between phylogenetic and functional distances for these SAGs (Fig. 3c), with the slope and *R*^2^ values differing between C- depleted and C-enriched soils. This relationship appears to be more pronounced in the C-depleted soils, suggesting that in C-depleted soils, microorganisms with different phylogenetic relationships tend to develop more distinct functions to cope with nutrient scarcity and competitive pressure. In contrast, the observed correlation was weaker in the C-enriched soils, which may be related to a higher degree of functional redundancy in the genes associated with organic carbon cycling in microorganisms in the C-enriched soils.

### Higher metabolic interaction potential in C-enriched soil bacterial community

To achieve a deeper analysis of microbial community features at the single-cell level, we integrated scCS-seq with metagenomics. This combined approach quantifies the abundance of individual microbes (SAGs) within a community and uncovers co-occurrence patterns and reconstructs metabolic networks. With this approach we first generated abundance profiles of microbial communities in relation to abiotic environmental factors. Principal coordinates analysis showed a clear structure of community genome assemblages by sampling site and carbon availability (Fig. S5). Genome-scale community composition was primarily influenced by pH, total carbon (TC), and available phosphorus (AP). To disentangle the effect of carbon availability from co-varying environmental factors, we performed PGLS analyses incorporating soil pH and AP as covariates and accounting for phylogenetic relatedness among SAGs based on the GTDB reference tree (Table S3). Carbon enrichment remained a significant predictor of genome size and GC content after controlling for these variables, and variation partitioning further showed that SOC explained an independent fraction of community variation (~8%) after accounting for pH and AP (Table S3).

**Figure 4:**
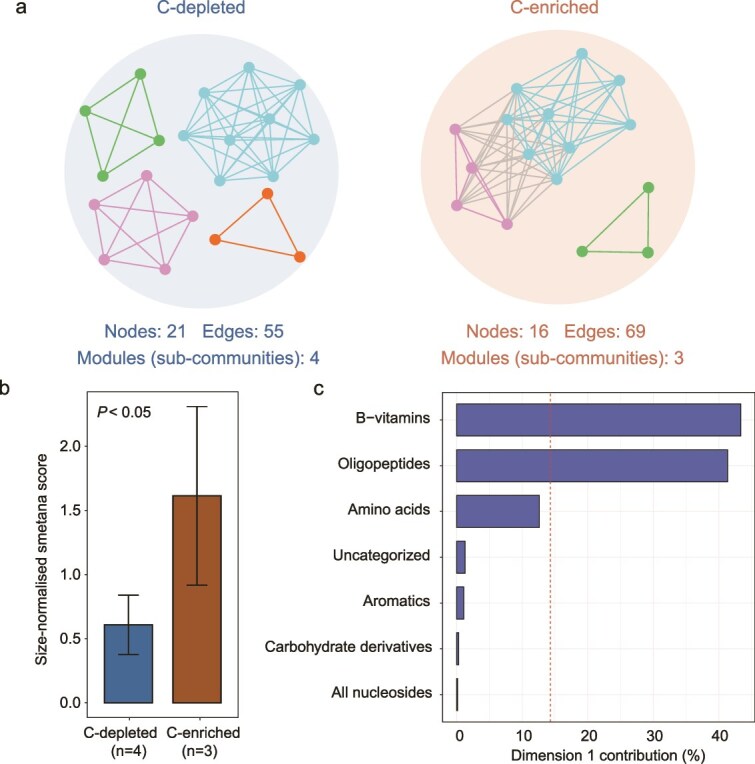
Genome co-occurrence network and metabolic interaction potential within soil bacterial communities. (a) Genome co-occurrence network for the C-depleted and C-enriched soils. These networks are visualized as a set of nodes, edges, and modules, where nodes represent SAGs, and edges denote potential interactions between these SAGs, and modules represent each sub-community. Edges represent robust microbial associations that pass dual thresholds—statistical significance (*P <* .05) and association strength (|r| > 0.6). Different colors represent different modules. Different modules in the network are considered as a sub-SAG community. (b) Bar plot compares the size-normalized SMETANA scores between the C-depleted and C-enriched soils. (c) The metabolic categories driving the potential for exchanges in the C-depleted and C-enriched soils bacterial communities.

Further, we inferred microbial co-occurrence networks by SAG abundances to investigate the interactions and relationships among SAGs within the community. We observed that the C-depleted network consisted of 21 nodes connected by 55 edges, which were organized into 4 distinct sub-communities (Fig. 4a). The C-enriched network, despite consisting of only 16 nodes, exhibited a much higher number of 69 edges and organized into 3 distinct sub-communities (Fig. 4a). Null model analyses further showed that network modularity was significantly higher than random expectations in both C-enriched (SES = 3.309, *P <* .001) and C-depleted soils (SES = 11.849, *P <* .001) (Table S4), confirming that the observed modular organization represents non-random biological structure. To evaluate the influence of unequal sample sizes, we performed a rarefaction-based resampling analysis (1000 iterations), which consistently showed higher network density in C-enriched communities than in C-depleted communities (permutation test, *P <* .001), indicating that the observed differences are robust to sample size variation (Fig. S6). These results indicate that C-enriched soils harbor more tightly interconnected microbial networks. To assess whether this increased network connectivity corresponds to enhanced metabolic interactions, we calculated the metabolic interaction potential for SAGs within each sub-community using SMETANA. The C-enriched soils exhibited a significantly higher size-normalized SMETANA score (*P <* .05) compared to the C-depleted soils (Fig. 4b). To account for the sensitivity to genome completeness, we applied strict genome quality thresholds (≥70% completeness and ≤5% contamination) to select 20 high-quality genomes (15 from the C-enriched group and 5 from the C-depleted group). Our results show that the C-enriched group continued to exhibit higher average SMETANA scores than the C-depleted group (Fig. S7). These results suggest that microbial communities in C-enriched soils may have a greater potential for metabolic interactions, consistent with predictions from SMETANA modeling.

To further explore and identify molecular mechanisms driving these patterns of predicted metabolic interactions, we analyzed the metabolic exchanges in the genome-scale community described above. In the PCA plot, the C-depleted soils community is concentrated in the central region, indicating a higher consistency in their metabolic functions, with lower variability in metabolite exchange and relatively lower diversity in metabolic interactions (Fig. S8). In contrast, the C-enriched soils community displays a broader distribution, suggesting a higher diversity in their metabolic exchanges (Fig. S8). The first dimension of co-variation (Dim1) highlighted B-vitamins, oligopeptides, and amino acids as the metabolic categories most preferentially exchanged within microbial communities (Fig. 4c). We then related the PCA contributions of these key categories to their raw SMETANA scores and evaluated differences between treatments. Amino acids and aromatic compounds were predicted to have significantly higher exchange potential in the C-enriched group (log₂ FC =1.42, *P* = .016 and log₂ FC =0.76, *P =* .010, respectively) (Table S5). In contrast, although oligopeptides accounted for a substantial proportion of the overall PCA variation (41.3%), their exchange potential did not differ significantly between treatments (Table S5), indicating comparably strong oligopeptide exchange under both carbon conditions. B-vitamins contributed most strongly to PCA variation but were detected exclusively in the C-enriched group (Table S5), suggesting that vitamin-mediated exchanges may represent a distinctive metabolic feature of carbon-rich environments.

To strengthen the model’s findings, we have performed a co-cultivation experiment followed by LC–MS metabolomics to validate the predicted metabolic exchanges between the C-enriched and C-depleted groups. Our analysis indicated that the Smetana score for Uracil is significantly higher in the C-depleted group (*P <* .05) (Fig. S9a). The abundance of Uracil in the supernatant is also significantly higher in the C-depleted group (*P <* .05) (Fig. S9b), consistent with the Smetana score results. This validates the model’s prediction that Uracil is exchanged at a higher frequency in the C-depleted group, reinforcing the observed metabolic interaction. The Smetana score for Glucose 3-phosphate is significantly higher in the C-enriched group (*P <* .05) (Fig. S9c), indicating a stronger predicted cross-feeding potential for this metabolite in the C-enriched group. The abundance of Glucose 3-phosphate is significantly higher in the C-enriched group (*P <* .05) (Fig. S9d), which aligns with the higher Smetana score for this metabolite. The result is consistent with the model’s prediction, indicating that Glucose 3-phosphate is more abundant in the C-enriched group under our experimental conditions and could potentially contribute to metabolic cross-feeding in carbon-rich environments.

## Discussion

Carbon availability plays a critical role in soil microbial communities, influencing not only microbial metabolic activity and trophic metabolic interactions but also having profound effects on the stability of community structure and function [1–3]. Here, we show that long-term organic carbon enrichment drives the evolution of soil microbial community composition and functional traits by altering the availability of carbon sources, particularly enhancing carbon degradation and transformation capabilities. Under C-enriched conditions, microbial communities are predicted to exhibit stronger carbon metabolism and more complex metabolic interaction networks, which could have profound implications for soil ecosystem function and agricultural sustainability.

In this study, we introduce a method based on “single-cell Cell Sorting and Sequencing” (scCS-seq) for isolating individual microorganisms from soil samples. The scCS-seq system offers a sorting throughput comparable to that of traditional fluorescence-activated cell sorting techniques [39], but with significant advantages for fragile microorganisms or sensitive samples. By eliminating the need for fluorescent or isotopic labeling, this method minimizes the potential impact on microbial viability, enabling gentler sample handling and maintaining high sorting efficiency and preserving microbial activity. This approach has also effectively isolated individual microorganisms in wastewater [8] and gut studies [40], facilitating genome acquisition and providing valuable opportunities for advancing single-cell microbiology research. Our scCS-seq results indicate that the proteobacteria phylum exhibited the highest representation in the SAG dataset, highlighting its central role in soil microbial ecology. This finding is consistent with our previous results [2]. Under C-enriched conditions, SAGs were distributed across multiple phyla, reflecting greater microbial diversity and a broader phylogenetic spectrum. In contrast, under C-depleted conditions, SAGs were concentrated within specific phyla, which may be due to reduced microbial diversity and increased phylogenetic convergence in C-depleted soils [41]. These findings suggest that carbon availability in soil not only influences microbial abundance but also has profound effects on community structure and evolution, particularly in terms of diversity and phylogenetic breadth. In terms of microbial life history strategies, SAGs exhibited larger genome sizes and higher coding sequences (CDS) in the C-enriched soils, suggesting greater metabolic diversity and adaptability to exploit a wider range of environmental niches and resources [2, 42]. Larger genomes typically possess a wider array of functional genes and greater metabolic versatility, allowing microorganisms to adapt to a wider range of environmental conditions and utilize diverse substrates [43]. However, smaller genomes may pose ecological risks. For instance, when microorganisms with smaller genomes are dispersed into new environments or face environmental disturbances, they may be outcompeted by microbes with larger genomes [44].

For bacteria, the number of genes in most high-level functional categories (such as KEGG functional hierarchies) has been shown to scale as a power-law to the total number of genes in the genome [45]. In bioecology, there are many scaling relationships and laws, such as Kleiber’s law, which describes how metabolic rate scales with body mass in birds and mammals [46]. And these genomic scaling laws have been shown to be conserved across microbial clades and lifestyles, supporting the observation that they are universally shared by all prokaryotes [47]. The C-depleted soils exhibit smaller residuals, indicating that they more closely follow the genome scaling law. This suggests that microorganisms in the C-depleted soils may have adapted to the carbon- depleted environment through genomic streamlining [48]. SAGs exhibited higher GC content in the C-depleted soils, which likely confers a survival advantage in low-nutrient environments. Specifically, high GC content is associated with greater genomic stability and resilience, because GC-rich DNA is more thermally stable and less prone to denaturation [43]. This increased stability may allow microorganisms to better withstand the stresses of extreme conditions, such as microbial communities in bare soils with higher GC content are better adapted to oligotrophic environments and can endure higher levels of radiation and temperature [43].

In contrast, SAGs exhibited shows a more diverse and abundant CAZymes in the C-enriched soils, indicating microbial communities in these environments are well-equipped to degrade a wider variety of substrates, leading to more efficient nutrient cycling [2]. GT genes (GT83 and GT44) are represented higher in C-enriched soils. These genes from the glycosyltransferase families are primarily involved in carbohydrate biosynthesis, including the modification of polysaccharides and glycoproteins, thereby contributing to the enhanced biosynthetic capacity of microorganisms [49]. This functional enrichment is consistent with our stable isotope probing results, which showed correspondingly higher microbial biomass and turnover rates in C-enriched soils (Table S6). These findings suggest that organic carbon inputs promote microbial strategies emphasizing growth and biosynthesis. Conversely, the C-depleted soils show an enrichment of AA3. This enzymes belong to the glucose–methanol–choline (GMC) oxidoreductase family and are oxidative enzymes. They are typically involved in the degradation of complex aromatic polymers, such as lignin, by generating hydrogen peroxide and other reactive species that act synergistically with lignocellulose-degrading enzymes [50]. This may facilitate the efficient degradation of complex organic carbon, promoting microbial access to essential nutrients and supporting microbial survival in nutrient-limited environments [51]. Such specialization to use complex organic compounds can increase the competitive advantage of microorganisms in C-depleted soils [52]. This suggests that the C-depleted soils foster microbial strategies focused on resource acquisition.

The positive correlation between the phylogenetic distance and functional distance among SAGs indicates that microorganisms with greater evolutionary divergence tend to exhibit more pronounced functional differences. This supports the idea that taxonomic diversity is linked to functional diversity [53]. This correlation is observed in both C-depleted and C-enriched soils, highlighting that the relationship between evolutionary divergence and functional diversity is a general characteristic of microbial communities [54]. The correlation between evolutionary divergence and functional capacity is more pronounced in C-depleted soils. This suggests that the increased functional divergence among phylogenetically distinct microorganisms in these environments may be a response to specific nutritional constraints and competitive pressures present in such conditions [52]. In contrast, the observed functional redundancy was higher in the carbon enriched soils, particularly regarding functions related to organic carbon cycling. The presence of organic carbon in the soil likely leads to greater functional overlap or redundancy, enhancing resource use efficiency and reducing competition for limited nutrients. This functional redundancy suggests that long-term carbon enrichment may lead to more complex and resilient microbial communities, which enhances the carbon cycling capacity of soil microorganisms.

Our results also revealed a clear structure of soil microbial community genome assemblages and identified abiotic and biotic factors driving genome-scale community composition. Although long-term fertilization inevitably alters multiple soil physicochemical properties, including pH [55], phosphorus availability, and SOC, redundancy analysis indicated that both pH and SOC are important environmental factors driving variations in microbial community structure, consistent with previous studies in long-term fertilization systems (Fig. S10). Under organic fertilization regimes, these two variables are often highly correlated. SOC exhibits stronger and more ecologically meaningful explanatory power in multivariate models, indicating that carbon availability is the primary factor driving changes in microbial community structure [41]. Biological factors such as competition, parasitism, and mutualism are expected to play an equally significant role in shaping microbial communities [56], although these interactions are more challenging to study in natural environments. Microbial association networks provide a valuable framework for representing potential biological interactions, capturing novel properties that emerge from these interactions [13, 16, 57]. However, it is important that the identified correlations reflect potential associations rather than direct causal relationships, further experimental validation is required to confirm direct microbial interactions. The microbial networks in C-enriched soils exhibit a greater number of edges, indicating more complex interactions within the microbial community. This complexity likely reflects higher levels of microbial cooperation and/or competition, leading to greater functional diversity and stability within the community [14, 15].

To advance beyond co-occurrence correlation-based approaches and develop a more mechanistic understanding of soils microbial community functioning, we sought to model the community metabolism of co-occurrence soil microbial genomes (SAGs). It is important to clarify that SMETANA scores quantify the predicted metabolic interdependencies among community members, with higher scores reflecting greater potential for metabolite exchange and cross-feeding. Although unequal SAG numbers across treatments could potentially bias network-based comparisons, this effect is explicitly accounted for by the size-normalized SMETANA framework, in which interaction potential is scaled by sub-community size. As expected, microorganisms in C-enriched soils exhibit a higher potential for metabolic interactions (i.e. higher size-normalized SMETANA scores). This is likely due to the greater diversity and complexity of organic matter in these environments. Such interactions may involve the sharing of metabolic byproducts or division of labor among different microbial species, thereby enhancing the overall cooperation and functionality of the community [2]. In contrast, under carbon-limited conditions, microorganisms allocate more energy to produce various extracellular enzymes to break down organic compounds, enabling them to access energy and nutrients, similar to the previously mentioned findings [51]. This reflects that interactions in C-depleted soils are likely characterized by scramble competition (exploitation competition), where microbes rapidly utilize limited resources by secreting nutrient-acquiring enzymes [52]. Sensitivity analyses applying more stringent genome quality thresholds yielded consistent results, indicating that our main conclusions regarding carbon-associated differences in metabolic interaction potential are robust to stricter genome quality filtering.

We identified specific metabolites predicted to be preferentially exchanged in C-enriched and C-depleted soils. Our results indicate that oligopeptide exchange does not differ significantly between treatments, despite contributing substantially to overall variation, suggesting that oligopeptide exchange represents a stable, baseline form of metabolic interaction. Under C-enriched conditions, the overall metabolic interaction pattern shifts toward an exchange network centered on amino acids and aromatic compounds. The prominence of predicted amino acid exchanges suggests that synthetic interactions may play an important role in supporting cooperative growth among microorganisms under carbon-enriched conditions. Amino acids are vital for microbial growth and metabolism, serving as essential building blocks for proteins needed for cellular functions and reproduction. In C-enriched soils with abundant organic matter, microorganisms often exchange amino acids through mutualistic relationships and cross-feeding, because amino acids are readily usable and support cooperative metabolic processes [58–60]. Amino acids serve as a potential evolutionary optimizing strategy to reduce biosynthetic burden and promote cooperative interactions. This observation is in support of the black queen hypothesis [61, 62], which proposes that some organisms offload costly metabolic functions to others in a cooperative manner for mutual benefit.

The cross-feeding predictions made by SMETANA represent potential interactions, rather than direct evidence of actual cross-feeding phenomena occurring in the system. Therefore, independent experimental validation is essential to assess the biological relevance of these model-based predictions. Our co-culture experiment and metabolomic analysis demonstrate that the SMETANA model has predictive power in identifying key metabolites that mediate cross-feeding interactions within microbial communities. The consistency between SMETANA predictions and metabolite abundance measurements supports the hypothesis that carbon availability could be a primary driver of microbial cross-feeding and metabolic interactions, though further experimental validation is needed to confirm these interactions *in situ*. The higher metabolic potential for specific metabolites in both the C-enriched and C-depleted groups highlights the critical role of carbon status in shaping microbial community dynamics and suggests that these differences may reflect distinct metabolic strategies. Predicted metabolite exchange does not necessarily imply direct cross-feeding among living cells. Metabolites may also become available through alternative processes, including microbial cell turnover and phage-mediated lysis, which can release intracellular compounds into the surrounding environment. The SMETANA framework infers potential metabolic dependencies based on genome content, but it does not distinguish among these underlying mechanisms of metabolite release. Moreover, in carbon-enriched soils, the observed increase in metabolite abundance and the predicted enhancement of metabolic dependencies may result from multiple co-occurring factors, including active metabolic interactions, elevated biomass turnover rates, and viral predation. These processes are not mutually exclusive and may collectively shape the observed patterns of predicted metabolic interactions. Future studies integrating stable isotope probing, viral metagenomics or viromics, and comprehensive community-level analyses will be essential to elucidate the relative contributions of these mechanisms. Here, our findings remain model-dependent and are limited to a subset of metabolites under controlled conditions, given the vast diversity of metabolites and exchange interactions in natural systems. Future research that includes a broader range of metabolites and environmental conditions could further enhance the accuracy of these predictions and provide a more comprehensive understanding of microbial metabolic networks.

Although the SAG approach provides unique insights into uncultivated microbial genomes, due to limited genome coverage and incompleteness, this method is more suitable for genomic-scale community analysis. The number of SAGs obtained in this study was limited, this is a common challenge in single-cell genomics given the technical complexity of the approach. Even a modest number of SAGs can provide unique insights that cannot be captured by MAGs, including access to rare lineages, strain-level heterogeneity, and different metabolic traits. This study also advances beyond prior metagenomic-only approaches by integrating single-cell cell sorting and sequencing, which provides high-resolution, strain-level genomic information into microbial communities, facilitating a more detailed understanding of microbial metabolic potential and functional interactions, particularly in the study of uncultivated taxa and low-abundance populations. Our dataset may not be fully representative of the entire community, but rather serves as a complementary perspective to community-level profiles, offering insights into aspects of microbial diversity and cross-feeding potential that would otherwise remain hidden. Future studies should focus on expanding SAG coverage and improving genome completeness. Linking microbial community characteristics and cross-feeding network features to soil carbon sequestration, nutrient cycling, and even crop productivity will help reveal the specific mechanisms through which microbial communities contribute to soil health and agricultural sustainability. Overall, our framework, which combines scCS-seq with metabolic modeling, provides a critical theoretical foundation for better understanding the role and mechanisms of microbial interactions in driving ecosystem functions.

## Conclusion

Our study explores the genomic and functional characteristics of soil microorganisms under different carbon availability conditions from a single-cell microscopic perspective. In C-enriched soils, the enrichment of GT genes (GT83 and GT44) enhances microbial biosynthetic capacity, promoting growth and community turnover. C-depleted soils show an increase in AA3 gene associated with catalyzing the oxidation of carbohydrates, reflecting a microbial strategy geared towards the efficient degradation of carbohydrates, facilitating nutrient access and survival in nutrient-limited conditions. Microbial genomes in C-depleted soils are more streamlined, whereas C-enriched soils exhibit greater functional redundancy, especially in carbon cycling. Using a metabolic modeling framework, we predicted potential metabolic interdependencies between microbial genomes, which were particularly pronounced in C-enriched soils. These findings suggest that microbial communities optimize their survival strategies through the sharing of resources and metabolic dependencies, which may represent a key mechanism shaping microbial community assembly in C-enriched environments. Our work identified the critical role of microbial functional traits in long-term organic inputs, which are essential for maintaining and stabilizing soil functions.

## Supplementary Material

wrag071_Supplementary This file includes: Figs S1–S10 and Tables S1 and S5.

## Data Availability

All data needed to evaluate the conclusions in the paper are present in the paper and/or the Supplementary Materials. All raw data used in this study are available in the NCBI, accession numbers PRJNA1214868. The analysis pipeline of this study has been uploaded to GitHub (https://github.com/qyz1688/Single-cell.git).
