# Supplementary material for: Single-cell assembled genomes predict enhanced bacterial metabolic cross-feeding potential in carbon-enriched soils
Source: ISME J. 2026 Mar 27;20(1):wrag071. doi: 10.1093/ismejo/wrag071 (PMC13123517; doi:10.1093/ismejo/wrag071)
Supplement: wrag071_Supplementary — This file includes: Figs S1–S10 and Tables S1 and S5. [file wrag071_supplementary.docx]

**Single-cell assembled genomes predict enhanced bacterial metabolic cross-feeding potential in carbon-enriched soils**

Hanyue Guo ^a, 1^, Qicheng Xu ^a, 1,^ *, He Zhang ^a^, Yizhu Qiao ^a, b^, Yang Song ^b,^ *，Yinghua Duan ^c^, Ning Ling ^a^^, d^, Qirong Shen ^a,^ *

*a, Jiangsu Provincial Key Lab for Organic Solid Waste Utilization, National Engineering Research Center for Organic-based Fertilizers, Jiangsu Collaborative Innovation Center for Solid Organic Waste Resource Utilization, Nanjing Agricultural University, Nanjing, 210095, China.*

*b, Plant–Microbe Interactions, Institute of Environmental Biology, Department of Biology, Science4Life, Utrecht University, Utrecht, the Netherlands*

*c, State Key Laboratory of Efficient Utilization of Arid and Semi-arid Arable Land in Northern China / Institute of Agricultural Resources and Regional Planning, Chinese Academy of Agricultural Sciences, Beijing, 100081, China*

*d, Centre for Grassland Microbiome, State Key Laboratory of Grassland Agro-ecosystems, College of Pastoral Agricultural Science and Technology, Lanzhou University, Lanzhou, 730020, Gansu, China*

**Running title:** Single-cell genomes in carbon-rich soils

^1^ Hanyue Guo and Qicheng Xu contributed equally to this paper.

***Corresponding author:**

*Qicheng Xu; E-mail address:* [*qichengxu@njau.edu.cn*](mailto:qichengxu@njau.edu.cn)

*Yang Song; E-mail address: y.song1@uu.nl*

*Qirong Shen; E-mail address:* [*shenqirong@njau.edu.cn*](mailto:shenqirong@njau.edu.cn)

**Address:** Qicheng Xu, College of Resources and Environmental Sciences, Nanjing Agricultural University, No. 666 Binjiang Avenue, Jiangbei New District, Nanjing, Jiangsu, 211800, China

Yang Song, Plant-Microbe Interactions, Department of Biology, Science4Life, Utrecht University, Padualaan 8, 3584 CH, Utrecht, The Netherlands

Qirong Shen, College of Resources and Environmental Sciences, Nanjing Agricultural University, No. 666 Binjiang Avenue, Jiangbei New District, Nanjing, Jiangsu, 211800, China

**Supplementary Materials Methods**

**Sample information**

We collected soil samples from four different locations in China where chemical fertilizers and organic fertilizers were applied. Location: experimental field (126.6°E, 45.7°N) in Harbin, Heilongjiang Province since 1980; Laiyang, Shandong (120.7°E, 36.9°N) since 1978; Suixi, Anhui (116.7°E, 33.6°N) since 1981; Location: experimental field in Nanchang, Jiangxi (116.4°E, 28.3°N) since 1986; We then performed metagenomic and single-cell sequencing on these soil samples.

**Measurement of soil microbial biomass**

Soil microbial biomass was measured by chloroform fumigation-incubation method. Specifically, the 35 g soil sample was weighed in an aluminum box, placed in a vacuum desiccator, and killed with chloroform for 24 hours; At the same time, a batch of soil samples of the same weight was weighed into the leaching bottle and placed in the dark for 24 h. Immediately after the end of fumigation, all the soil samples were transferred from the aluminum box to the extraction bottle, and 50 mL K_2_SO_4_ solution (0.5 mol/L) was added. After shaking at 200 rpm/min for 30 min, the soil samples were filtered (or first centrifuged and then filtered), and the filtrate was diluted 20 times, passed through a 0.45 μm filter membrane, and determined on a TOC meter.

$$MBC(mg/kg)=\frac{\left（ P(fumigation)-P(no\_fumigation) \right）\cdot V\cdot dilution ratio*1000}{m*Kec /Ken}$$

P (fumigation): the concentration of total carbon in the extract of the fumigated sample (mg·L^-1^); P (no-fumigation): the concentration of total carbon in the extract of the unfumigated sample (mg·L^-1^); V: extraction volume (L); m: the soil sample was converted to the dried soil weight (g); the KEc conversion coefficient of MBC was 0.38.

**Measurement of soil microbial turnover**

Soil microbial turnover was measured by ^18^O isotope labeling method. Specifically, two treatments were set up for each soil sample (one for natural ^16^O abundance control and one for ^18^O labeled sample). Two 2 g samples were placed in 15 mL glass bottles sealed with rubber plugs, and the equal volume of ^16^O water was used as the natural water-corrected ^16^O abundance control treatment for unlabeled samples. The soil water holding capacity of 20 at% ^18^O enrichment was used as ^18^O labeled sample when the addition of ^18^O water reached 60%. The cells were incubated for 24h at 25° C and 65% humidity in the dark. Microbial DNA from each sample after 24 h incubation was extracted using the FastDNASPIN kit for soil and quantified using the Quant-iT PicoGreen dsDNA reagent (LifeTechnologies). After that, each DNA fraction extract was pipetted into a silver cup and dried at 40 to 60° C before abundance ^18^O and total O content were determined using IMS-TC/EA.

$${DNA}_{produced}=O_{total}*\frac{{at\%}_{excess}}{100}*\frac{100}{{at\%}_{label}}*\frac{100}{31.21}$$

$Turnover=\frac{{DNA}_{produced}*24*365}{{DNA}_{content}*t}$

O_total_ (ug) is the total amount of oxygen in the DNA sample; at%_excess_ is the difference in the abundance of ^18^O between samples with and without ^18^O. at%_label_ is the background abundance of ^18^O in the soil sample: 31.21% is the mass percentage of oxygen atoms in the DNA molecule.

**Bacterial strains used in co-cultivation experiments**

The bacterial strains used in the co-cultivation experiments are laboratory-stored, isolated from soils. The strains used in the carbon-enriched treatment include *Pseudomonas*, *Sphingomonas*, *Sphingopyxis*, *Sphingobium*, and *Edaphobacter*. The strains used in the carbon-depleted treatment include *Pseudomonas*, *Paraburkholderia*, *Burkholderia*, *Sphingomonas*, *Sphingopyxis*, *Sphingobium*, and *Edaphobacter*.

**Metabolome analysis**

Chromatography: An ACQUITY UPLC HSS T3 column (100 mm×2.1 mm, 1.8 µm, Waters) was used for separation. The mobile phase consists of phase A (5 mmol/L ammonium acetate + 5 mmol/L acetic acid + water) and phase B (acetonitrile). Gradient elution conditions were set as follows: 0~0.8 min, 2% B; 0.8~2.8 min, 2% ~ 70% B; 2.8~5.0 min, 70% ~ 90% B; 5.0~5.5 min, 90% ~ 100% B; 5.5~7.5 min, 100%B; 7.5~7.6 min, 100% ~ 2% B;7.6~10.0 min, 2% B; The flow rate is 0.35 mL/min. The injection volume for each sample was 4 µL. The column oven was maintained at 40℃

Mass spectrometry：A high-resolution tandem mass spectrometer Orbitrap Exploris 120 (Thermo Fisher Scientific) was used to detect metabolites eluted form the column. Each sample was operated in both positive and negative electrospray ionization mode. ESI temperature is 350℃. The voltage is +3800 volts in positive ion mode and -3400 volts in negative ion mode. The sweep gas pressure of the ion source is 1 Arb, Gas 1 (Auxiliary gas) pressures set to 15 Arb, Gas 2 (Sheath gas) pressures set to 50 Arb. The mass spectrometric data were obtained with full scan and data-dependent acquisition (DDA) modes. In one acquisition cycle, the full scan acquisition range is 70-1050 Da, and the resolution is 60k, AGC target is set to Standard，Maximum IT is set to Auto. Then, the top 4 signal ions with a signal accumulation intensity of more than 5000 were selected from the full scan for DDA scanning, the DDA resolution is 15k, AGC target is set to Custom，Maximum IT is set to Custom. Dynamic exclusion is set to 4 s.

**Table S1 Soil physical and chemical properties at the four sites**

| Plots |  | pH | SOC (g/kg) | TN (g/kg) | AP (mg/kg) | AK (mg/kg) |
| --- | --- | --- | --- | --- | --- | --- |
| Jiangxi | C-enriched | 6.27 a | 11.73 a | 1.38 a | 204.9 a | 130.00 b |
|  | C-depleted | 4.40 b | 9.20 b | 1.07 b | 17.01 b | 162.67 a |
| Anhui | C-enriched | 7.78 a | 15.39 a | 1.88 a | 18.36 b | 110.33 b |
|  | C-depleted | 5.85 b | 12.95 b | 1.64 b | 47.01 a | 126.33 a |
| Heilongjiang | C-enriched | 6.66 a | 18.12 a | 1.79 a | 11.57 b | 93.00 b |
|  | C-depleted | 5.50 b | 16.96 b | 1.85 a | 87.80 a | 121.00 a |
| Shandong | C-enriched | 7.25 a | 17.01 a | 1.81 a | 364.83 a | 117.33 a |
|  | C-depleted | 6.84 a | 6.07 b | 0.69 b | 3.30 b | 63.67 b |

Notes: The letters “a” and “b” in the table indicate statistically significant differences (*P* <0.05) between the different carbon availability soils, carbon-depleted (C-depleted) and carbon-enriched soils (C-enriched), within the same plot.

| SAG_ID | Completeness (%) | Contamination (%) | SAG_ID | Completeness (%) | Contamination (%) |
| --- | --- | --- | --- | --- | --- |
| 17_18 | 71.12 | 3.48 | 4_15 | 80.87 | 9.31 |
| 17_9 | 68.15 | 5.50 | 14_2 | 64.67 | 3.25 |
| 18_18 | 69.91 | 4.00 | 15_18 | 82.76 | 7.11 |
| 25_22 | 71.59 | 4.81 | 24_14 | 77.94 | 3.84 |
| 21_18 | 69.88 | 2.98 | 16_18 | 77.38 | 1.66 |
| 1_19 | 60.47 | 2.62 | 15_9 | 71.27 | 7.79 |
| 2_19 | 60.54 | 2.40 | 13_2 | 74.04 | 3.36 |
| 22_18 | 68.95 | 4.13 | 16_1 | 72.87 | 6.75 |
| 3_14 | 78.87 | 6.10 | 9_4 | 67.23 | 4.44 |
| 5_15 | 70.76 | 6.41 | 10_4 | 67.99 | 3.24 |
| 4_14 | 78.91 | 8.99 | 24_7 | 80.06 | 7.90 |
| 6_15 | 67.93 | 9.63 | 15_17 | 71.58 | 5.50 |
| 5_11 | 75.69 | 8.70 | 5_6 | 74.13 | 8.40 |
| 18_9 | 73.68 | 7.91 | 24_13 | 75.26 | 7.11 |
| 23_15 | 80.78 | 8.36 | 16_17 | 74.86 | 7.17 |
| 21_10 | 70.88 | 1.38 | 19_12 | 86.59 | 5.77 |
| 21_2 | 60.76 | 1.98 | 20_12 | 87.97 | 4.92 |
| 1_18 | 95.19 | 3.27 | 9_1 | 86.18 | 2.90 |
| 22_10 | 72.90 | 2.37 | 7_20 | 84.85 | 3.71 |
| 2_18 | 96.01 | 6.40 | 19_13 | 85.43 | 2.71 |
| 1_11 | 75.54 | 2.62 | 19_3 | 91.81 | 3.49 |
| 1_4 | 69.44 | 4.99 | 10_1 | 84.85 | 3.13 |
| 2_4 | 69.16 | 5.93 | 22_1 | 58.34 | 3.09 |
| 1_6 | 61.23 | 9.99 | 8_20 | 87.94 | 4.33 |
| 2_11 | 74.82 | 4.07 | 20_3 | 93.86 | 4.43 |
| 2_6 | 61.82 | 7.23 | 21_6 | 91.23 | 5.40 |
| 8_17 | 52.17 | 1.88 | 21_1 | 55.51 | 4.51 |
| 24_5 | 70.78 | 3.12 | 11_10 | 94.67 | 9.82 |
| 3_15 | 79.96 | 8.48 | 22_6 | 98.25 | 4.86 |

**Table S2 The completeness and contamination of SAGs**

**Table S3 Effects of carbon enrichment and SOC on microbial genome traits and community variation**

| Analysis | Variable | Effect size | *t* value | *P* value |
| --- | --- | --- | --- | --- |
| PGLS | Carbon-enriched (genome size) | **755754** | 3.12 | **0.003** |
| PGLS | Carbon-enriched  (GC content) | **-0.33** | -2.32 | **0.024** |
| Variation partitioning | SOC | **8.37%** | – | **0.003** |

**Table S4 Network topology and null model validation of soil microbial communities**

| Metric | C-enriched Soil Network | C-depleted Soil Network |
| --- | --- | --- |
| Nodes | 16 | 21 |
| Edges | 69 | 55 |
| Null Model Comparison |  |  |
| Modularity (SES) | 3.309 *** | 11.849 *** |

**Table S5 Metabolite category analysis based on PCA contribution, fold change, FDR *P* values, and preference in carbon-enriched vs. carbon-depleted**

| Metabolite Category | PC1 Contribution (%) | log₂FC | FDR *P* value | Preference |
| --- | --- | --- | --- | --- |
| B-vitamins | 43.33 | NA | NA | Only in Carbon-enriched |
| Oligopeptides | 41.34 | 0.95 | 0.370 | No significant |
| Amino acids | 12.60 | **1.42** | **0.016** | **Carbon-enriched** |
| Uncategorized | 1.26 | **1.25** | **0.010** | **Carbon-enriched** |
| Aromatics | 1.07 | **0.76** | **0.010** | **Carbon-enriched** |
| All nucleosides | 0.12 | -0.99 | 0.052 | No significant |
| Carb-Derivatives | 0.29 | NA | NA | Only in Carbon- depleted |

**Table S6 Soil microbial biomass carbon and turnover rate**

| Plots | Fertilization | MBC (mg/kg) | Turnover (d) |
| --- | --- | --- | --- |
| Jiangxi | C-enriched | 204.8±0.8a | 238.8±0.06a |
|  | C-depleted | 170.6±0.4b | 218.2±0.09b |

Notes: The letters “a” and “b” in the table indicate statistically significant differences (*P* <0.05) between the different carbon availability soils, carbon-depleted (C-depleted) and carbon-enriched soils (C-enriched).


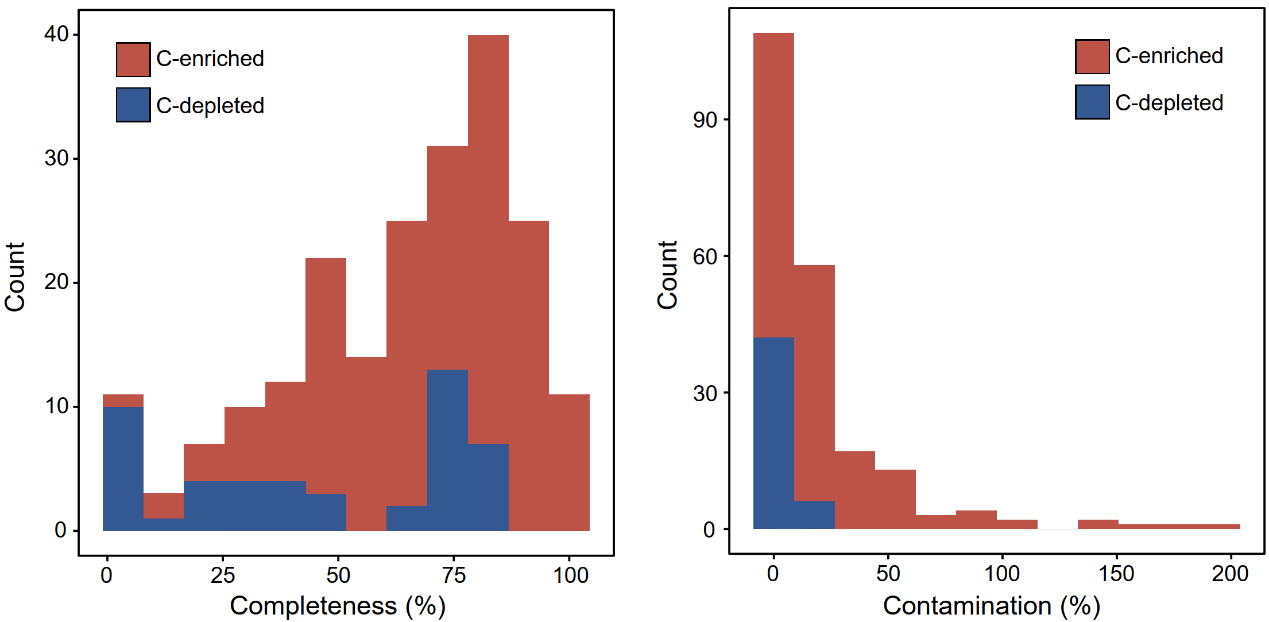
 **Fig. S1** Distribution of SAGs completeness and contamination across C-enriched and C-depleted groups.


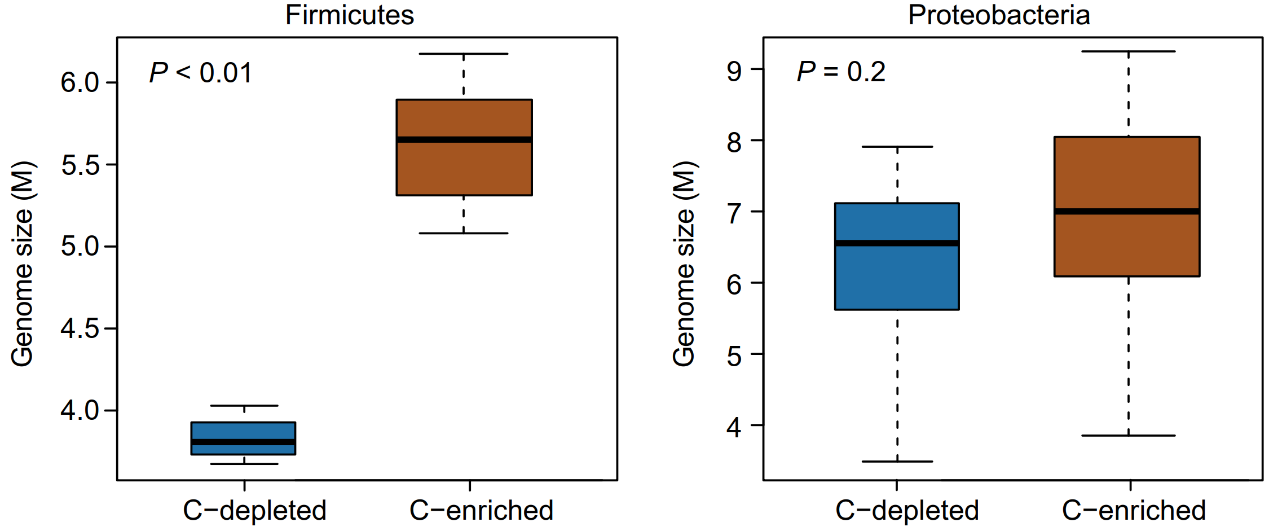


**Fig. S2** Genome sizes of Firmicutes (a) and Proteobacteria (b) under carbon-depleted and carbon-enriched soil conditions.


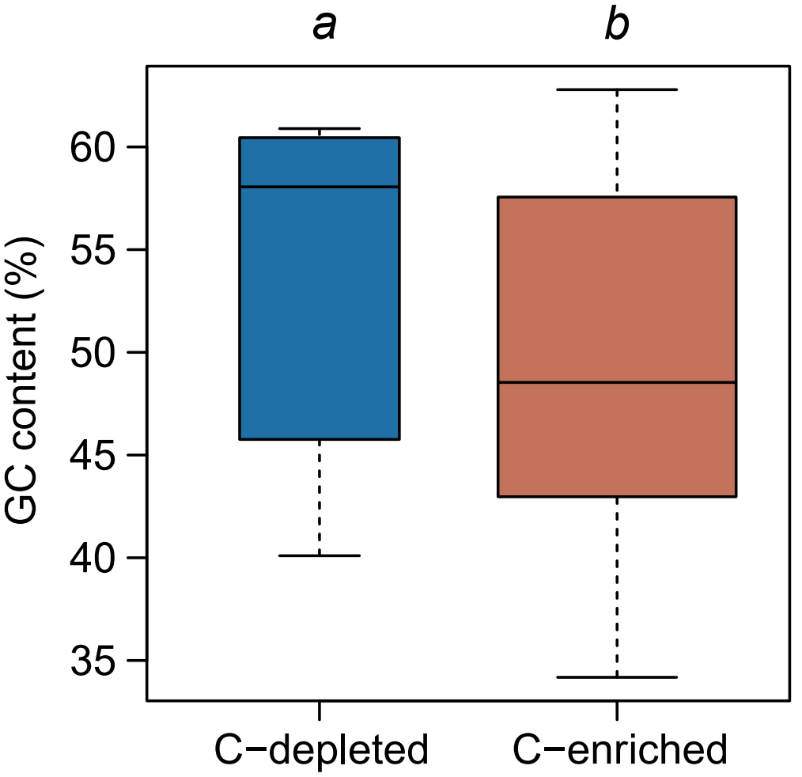
 **Fig. S3** The GC content of SAGs between the C-depleted and C-enriched soils.


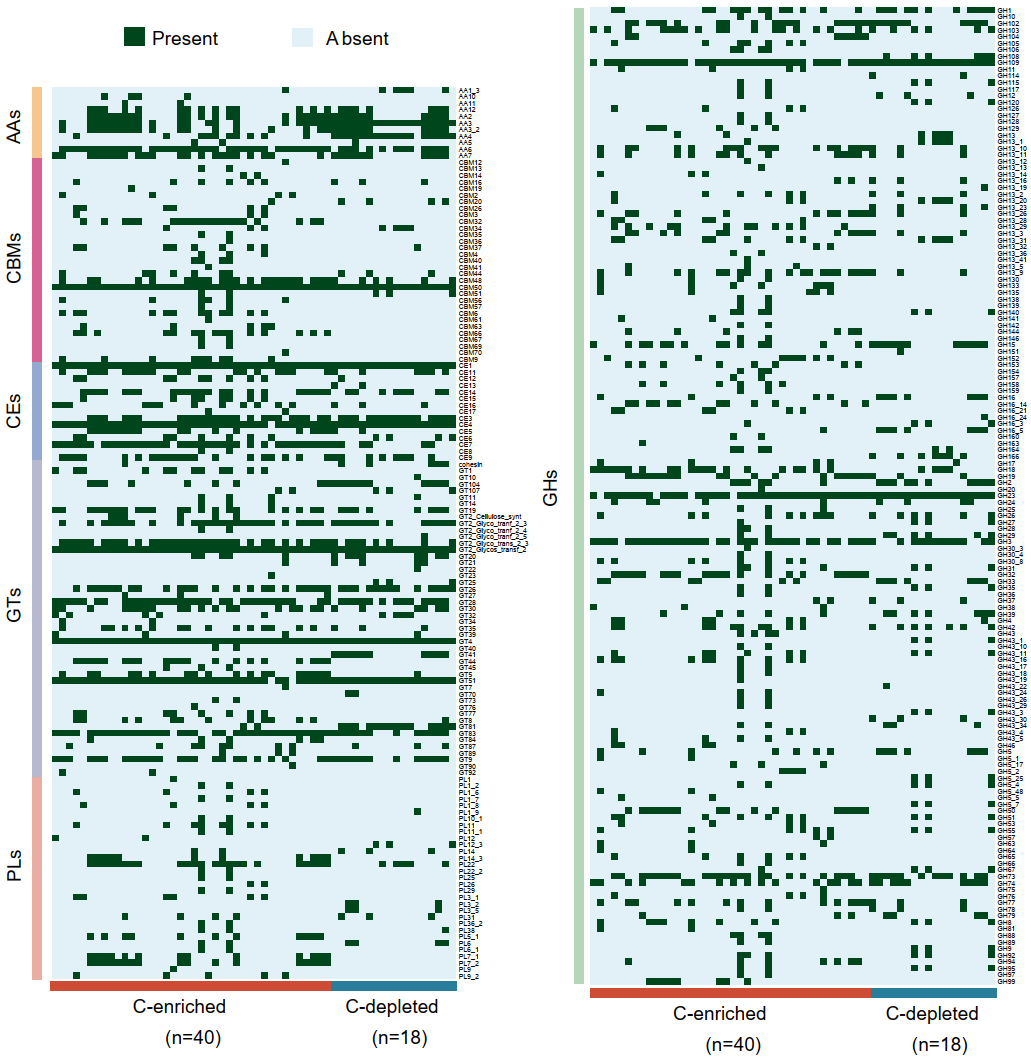


**Fig. S4** The heatmap showcases the presence and absence of various carbohydrate-active enzymes (CAZymes) in different SAGs, categorized into auxiliary activities (AAs), carbohydrate-binding modules (CBMs), carbohydrate esterases (CEs), glycoside hydrolases (GHs), glycosyl transferases (GTs), and polysaccharide lyases (PLs).


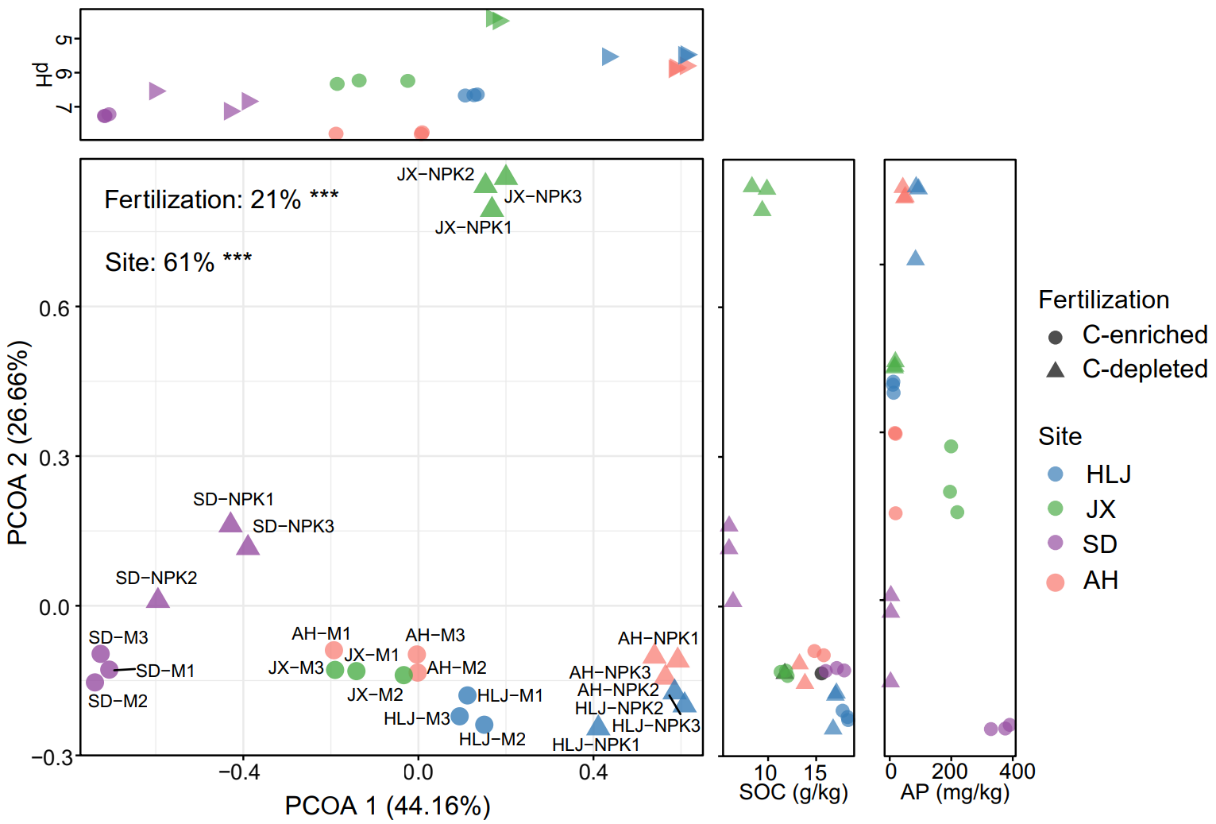


**Fig.S5** Principal Coordinates Analyses (PCoA) for genome-scale community abundances. Genome-scale community abundance (PCo1) is significantly associated with pH. PCo2 is associated with available phosphorus (AP) and soil organic carbon (SOC).


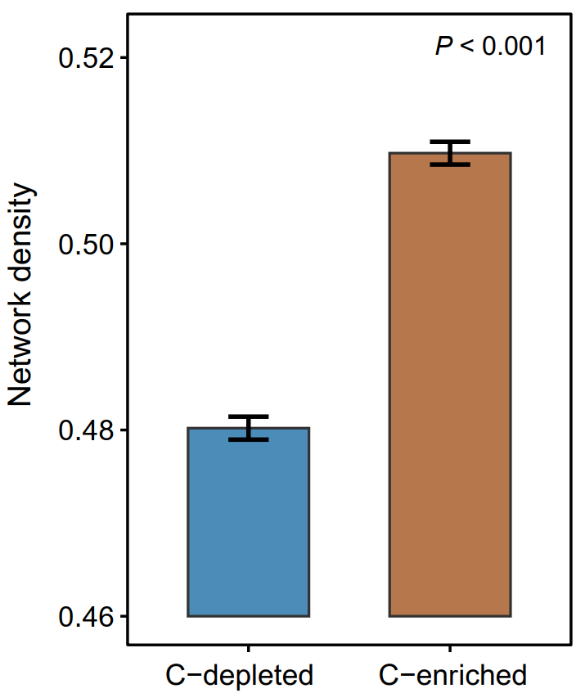


**Fig. S6** Comparison of network density between carbon-enriched and carbon-depleted communities. Network density was estimated through 1000 iterations of rarefaction analysis. Error bars represent 95% confidence intervals.


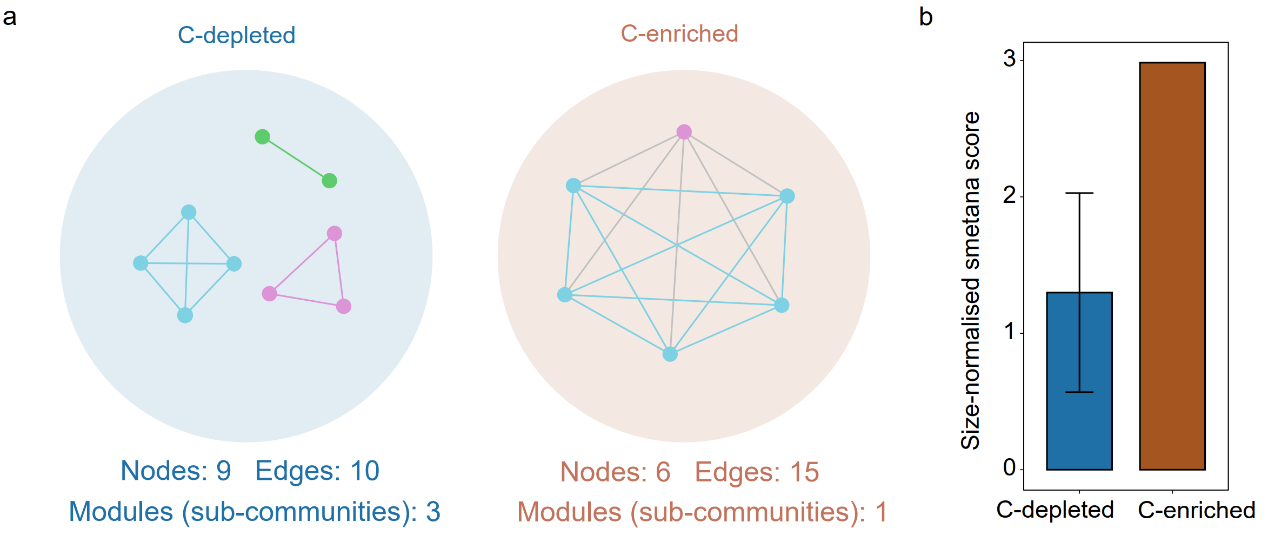


**Fig. S7** (a) Genome co-occurrence network for the C-depleted and C-enriched soils. These networks are visualized as a set of nodes, edges, and modules, where nodes represent high-quality SAGs (≥ 70% completeness and ≤ 5% contamination), and edges denote potential interactions between these SAGs, and modules represent each sub-community. Different colors represent different modules. (b) Bar plot compares the size-normalized SMETANA scores between the C-depleted and C-enriched soils.


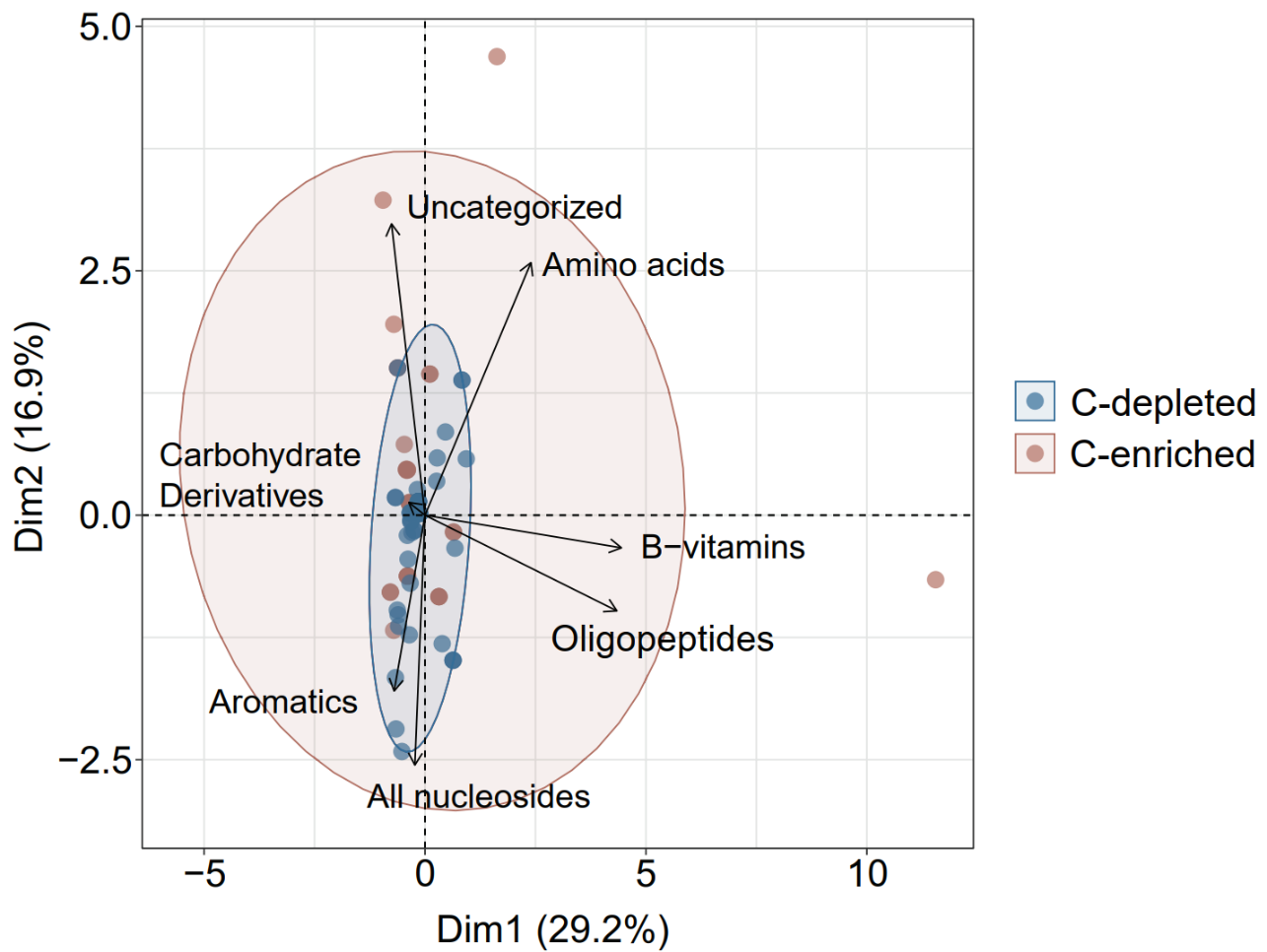


**Fig. S8** Principal Component Analysis (PCA) showing metabolic exchange potential across carbon depleted (blue triangles) and carbon enriched (red circles) soils communities. The arrows represent specific metabolic categories contributing to the variation, including amino acids, oligopeptides, aromatics, B-vitamins, and others. Coloured ellipses are visual aids to emphasize the distribution of points by categories. C-depleted and C-enriched communities exhibit distinct clustering patterns, indicating higher predicted metabolic exchange potentials in certain categories.


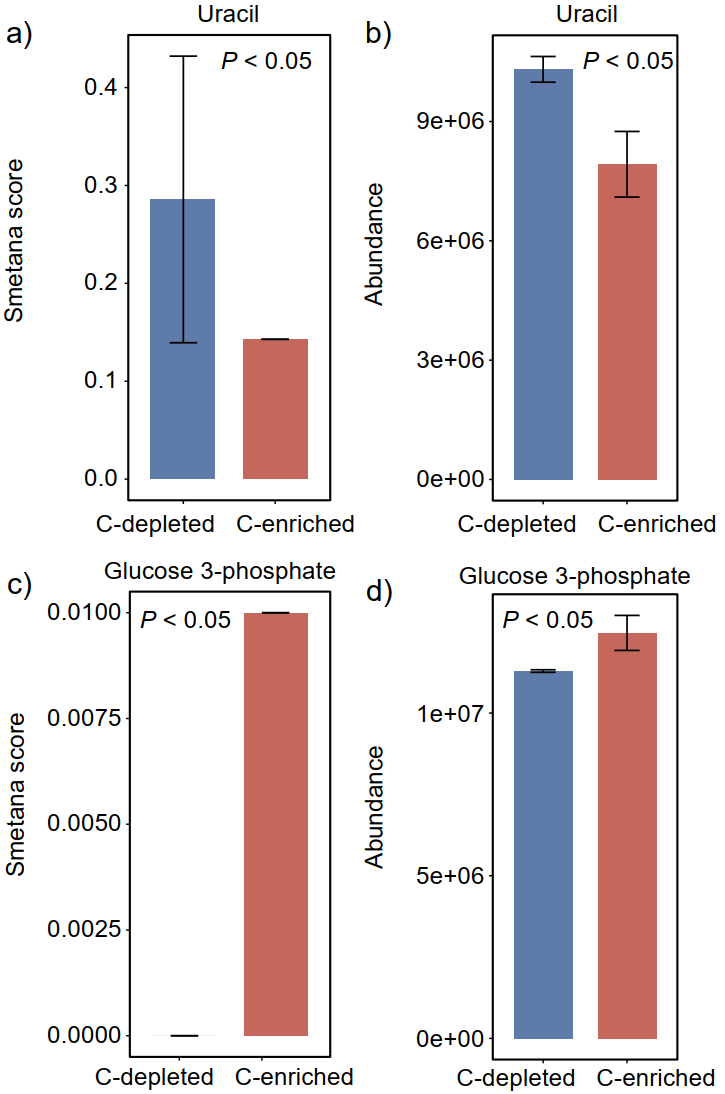


**Fig. S9** Comparison of Smetana scores and metabolite abundances between the C-depleted and C-enriched groups for specific metabolites. Panels (a) and (b) show the comparison of Smetana scores and metabolite abundances for Uracil, while panels (c) and (d) present similar comparisons for Glucose 3-phosphate between the C-depleted and C-enriched groups.


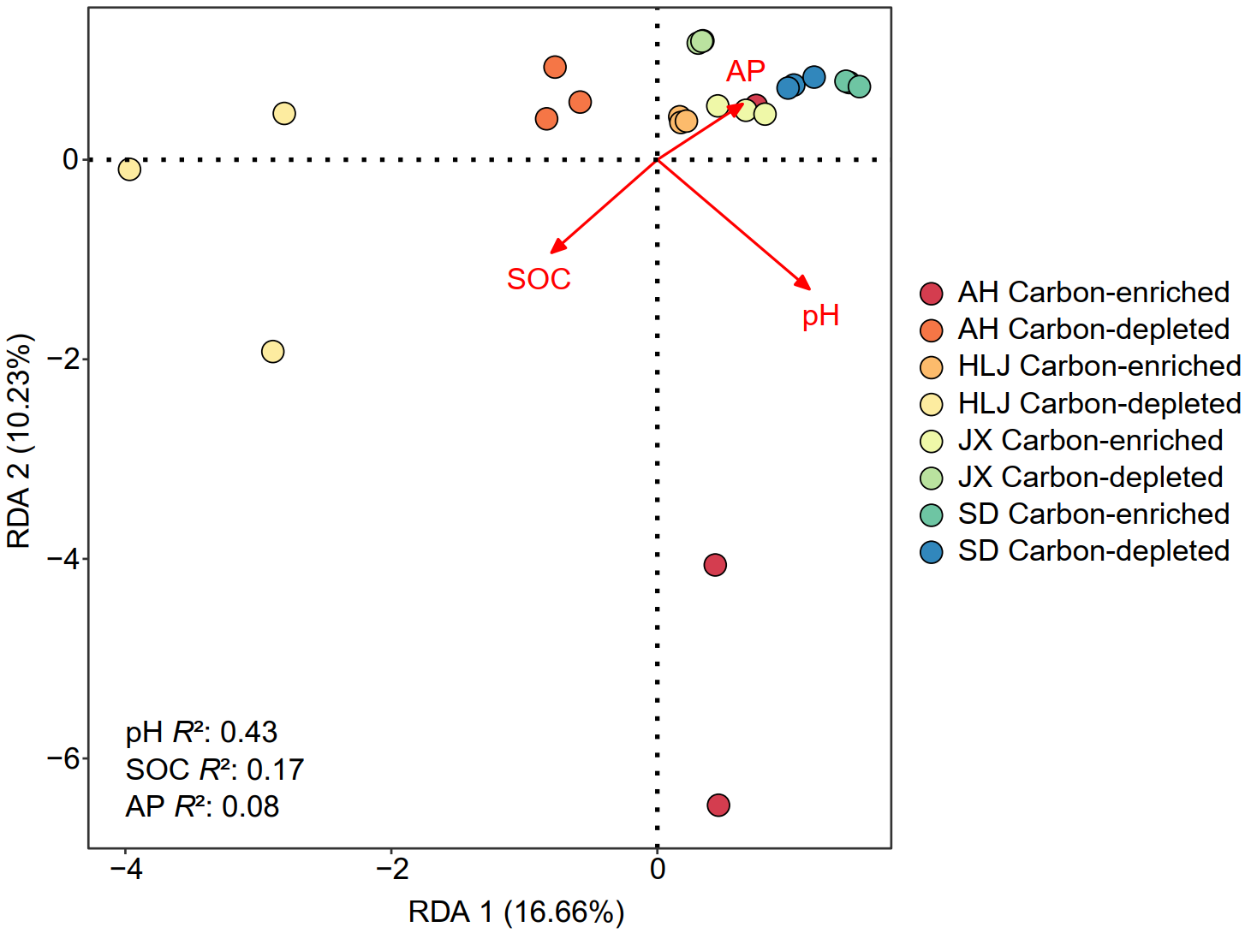


**Fig. S10** Redundancy analysis (RDA) of microbial community structure in response to soil carbon availability and other environmental factors.
